# Can “realist” randomised controlled trials be genuinely realist?

**DOI:** 10.1186/s13063-016-1407-0

**Published:** 2016-07-07

**Authors:** Sara Van Belle, Geoff Wong, Gill Westhorp, Mark Pearson, Nick Emmel, Ana Manzano, Bruno Marchal

**Affiliations:** Department of Public Health, Institute of Tropical Medicine, Nationalestraat 155, B-2000 Antwerp, Belgium; Nuffield Department of Primary Care Health Sciences, University of Oxford, Oxford, UK; Community Matters Pty Ltd, Mt Torrens SA, Australia; NIHR Collaboration for Applied Health Research and Care South West Peninsula (PenCLAHRC), Institute of Health Research, University of Exeter Medical School, Exeter, UK; School of Sociology & Social Policy, University of Leeds, Leeds, UK; Centre for Health, Technologies & Social Practice, School of Sociology & Social Policy, University of Leeds, Leeds, UK

**Keywords:** Randomized controlled trials, Realist evaluation, Scientific realism, Causation

## Abstract

In this paper, we respond to a paper by Jamal and colleagues published in *Trials* in October 2015 and take an opportunity to continue the much-needed debate about what applied scientific realism is. The paper by Jamal et al. is useful because it exposes the challenges of combining a realist evaluation approach (as developed by Pawson and Tilley) with the randomised controlled trial (RCT) design.

We identified three fundamental differences that are related to paradigmatic differences in the treatment of causation between post-positivist and realist logic: (1) the construct of mechanism, (2) the relation between mediators and moderators on one hand and mechanisms and contexts on the other hand, and (3) the variable-oriented approach to analysis of causation versus the configurational approach.

We show how Jamal et al. consider mechanisms as observable, external treatments and how their approach reduces complex causal processes to variables. We argue that their proposed RCT design cannot provide a truly realist understanding. Not only does the proposed realist RCT design not deal with the RCT’s inherent inability to “unpack” complex interventions, it also does not enable the identification of the dynamic interplay among the intervention, actors, context, mechanisms and outcomes, which is at the core of realist research. As a result, the proposed realist RCT design is not, as we understand it, genuinely realist in nature.

## Background

In October 2015, Jamal and colleagues [1] published a paper on realist randomised controlled trials (RCT), in which they present a detailed overview and guidance of how realist RCTs can be designed. Jamal et al. argue convincingly that RCTs “examine quite crude questions about ‘what works’ without explaining the underlying processes of implementation and mechanisms of action, and how these vary by contextual characteristics of person and place” (p. 1). They rightly point out the problems of generalisation of findings of RCTs of public health interventions, especially when the interventions are complex in nature. They also acknowledge that RCTs have difficulties in capturing the dynamic agency-structure interaction that shapes the implementation and the adoption by actors, as well as the outcomes of complex interventions. Yet the authors argue that realist RCTs address “these gaps while preserving the strengths of RCTs in providing evidence with strong internal validity in estimating effects” (p. 2).

We fully agree with the authors’ analysis of the shortcomings of RCTs of complex public health interventions. The RCT design has a number of limitations which make it not only difficult to apply to truly complex interventions but also incompatible with a realist approach to research, as we will describe herein. Although scholars like Blackwood et al. [2] have claimed that realism and RCT can coexist and while we appreciate the dedicated work of Jamal and colleagues, their article presents an important opportunity to continue the much needed debate about applied scientific realism—the foundation of the approach to realist research developed by Pawson and Tilley [3]—and the extent to which its logic becomes subverted when merged with post-positivist approaches to research design, such as the RCT.

Below, we explain how we identified three differences between the position of Jamal and colleagues and our own understanding of scientific realism. These are related to paradigmatic differences in the treatment of causation between post-positivist and realist logic: (1) the construct of mechanism, (2) the relation between mediators and moderators on one hand and mechanisms and contexts on the other hand, and (3) the variable-oriented approach to analysis of causation versus the configurational approach.

## Main text

In essence, Jamal et al. propose “realist RCTs” that use a technical process (moderator and mediator statistical analysis) to test hypotheses about observable phenomena. However, realism acknowledges that not all that matters can be observed (Table 1). In particular, realist research should concern underlying causal processes that lead to outcomes—so called mechanisms. While these may not be directly observable nor easily measurable, they matter; from a realist perspective, they are central to explaining causation.

**Table 1:** Key principles underpinning realist research [3, 11, 18, 19] and [20]

• Realism asserts a reality exists independently of the observer: both the material and the social worlds are “real”, at least in the sense that anything that can cause observable outcomes is itself real. • Knowing reality through science is unavoidably relative to the researcher: developing knowledge on reality is constrained by perception and cognition, and is socially constructed. Nonetheless, reality constrains the interpretations that are reasonable to make of it, meaning that it is possible to move gradually closer to an understanding that better reflects the reality under study. • According to realism, the world is differentiated and stratified, consisting not only of observable and measurable events, but also of structures, which have powers and liabilities capable of generating events. These structures may be present even where, as in the social world and much of the natural world, they do not generate regular patterns of events [20]. • Causality concerns not a direct relationship between two observable and discrete events, but a relationship between “the ‘causal powers’ or ‘liabilities’ of objects or relations, or more generally, their ways-of-acting of ‘mechanisms’” [20] and the outcomes of those mechanisms. • Context matters—a lot. Contextual conditions may have an influence on the implementation of the intervention, may or may not provide the necessary conditions for the mechanism that will be triggered and thus may have an effect on the observed outcome. An interaction is always present between context and mechanism. Context includes features such as social, economic and political structures; social policies; organisational context; participants; staffing; and geographical and historical context. • The interaction among intervention (strategies), actors, context and mechanism is what creates the intervention’s impacts or outcomes. • Since interventions work differently in different contexts and through different mechanisms, they cannot simply be replicated from one context to another and automatically achieve the same outcomes. Theory-based understandings about the influence of contexts on mechanism and resulting outcomes (i.e. “what works for whom, in what contexts, why and how” are, however, transferable. • Realist research is theory incarnate. It starts with a fragile theory and ends with a fallible model. Theory is to be understood as theories of the middle range, as defined by Merton [21], a bundle of hypotheses that can be tested empirically [22]. As such, these theories are expressed in such a way that they can be supported, refuted or refined against empirically derived data. Often, the term ‘programme theory’ is used to denote the starting point of a realist inquiry, which is one or more testable hypotheses that spell out how intervention strategies are expected to lead to the outcomes, for whom, in what conditions, how and why. • Realist research progresses through choosing methods that will provide the data needed to help “test” the initial programme theory in terms of effectiveness (*Did the programme achieve its goal?)* and of causal processes (*How did the observed results come about, in which context, why and for whom?*). Realist research is methodologically promiscuous, potentially using quantitative and qualitative data to test theories. • In summary, in the account of realism we propose here, outcome patterns do not occur directly because of the intervention strategies that are used. Instead, outcomes are caused by invisible mechanisms that are sensitive to context. The interactions and influences between context, mechanism and outcome may be explained by one (or more) middle-range theories. To develop, support, refute or refine these theories, both quantitative and qualitative data may be needed.	

### What is a mechanism?

The notion of mechanism is central to understanding disease aetiology and treatment. Mechanisms are also central in realist thinking, but these are conceptualized differently: interventions trigger mechanisms in specific contexts, and that leads to outcome patterns. The nature of mechanisms has long been a topic of discussion in realist circles [4–10], but wide agreement exists that “response to resources” is the defining feature of mechanisms in the work of Pawson and Tilley [3]. From a scientific realist perspective, intervention outcomes can be traced back to the resources offered and how stakeholders and participants respond to those resources. To put it another way, making and sustaining different choices requires a change in a participant’s reasoning (e.g. in their values, beliefs, attitudes or the logic they apply to a particular situation) and/or a change in the resources (e.g. information, skills, material resources, or support) they have available to them. Realism asserts that it is the interaction between “reasoning and resources” that underpins intervention outcomes [11]. As a consequence, interventions work in different ways for different people because they respond to resources offered by the intervention in different ways.

By their very nature, mechanisms are latent, invisible and sensitive to variations in context [12]. Mechanisms can play out at the level of individuals, groups, organisations and society. Ideas about mechanisms can be found in psychological, social, cultural, political and economic theories [6]. Thus “mechanism” in the realist sense does not equate to intervention components but rather to how the resources and opportunities created by the intervention are taken up (or not) by people in different contexts.

Jamal et al. define mechanisms as aspects of interventions. For instance, on p. 2, Jamal et al. write “The evaluator needs to hypothesise and test how the intervention theory of change interacts with context to enable (or disable) implementation, causal mechanisms and, ultimately, outcomes.” It is, however, not the “intervention theory of change” that interacts with context. Rather, scientific realism holds that interventions take place in specific contexts and address actors, who decide (or not) to change their behaviour, choices or decisions in response to the resources and opportunities offered by the intervention.

Mechanisms are also characterised by Jamal et al. as external treatments. On page 2, the authors write that “Realist evaluators have viewed interventions as ‘working’ by introducing mechanisms that interact with features of their context to produce outcomes.” This wrongly implies that mechanisms can be introduced into a situation and are thus external; scientific realism holds that mechanisms are not external factors but latent powers and capabilities, which are a function of the interaction between intervention resources and responses of participants.

All but one of the “mechanisms” presented by Jamal and colleagues in Table 2 (p. 8) are activities or implementation of actions. Only “increased commitment of disengaged students” would be considered a candidate mechanism in our understanding of realist research because it proposes a potential inherent power—“commitment”—that may or may not fire through students becoming more engaged. This mixing up of the concept of mechanism with that of intervention (strategy) is a common error, which overlooks the actual mechanisms at work [4, 13].

**Table 2:** Definitions of mechanism (adapted from Mahoney [14])

	Definition #1—“Variables” (successionist mode)	Definition #2—“Theory of change” (successionist mode)	Definition #3—Scientific realism (generative mode)
Definition	An intervening (set of) variable(s) that explain(s) why a correlation exists between an independent and dependent variable	Frequently occurring causal patterns that are triggered under generally unknown conditions and with indeterminate consequences. A mechanism explains by opening up the black box and showing the cogs and wheels of the internal machinery. It provides a continuous and contiguous chain of causal or intentional links between the *explanans* and the *explanandum*	An unobserved entity that, when activated, generates an outcome of interest
Analytical approach	Correlational analysis techniques, such as mediation analysis, are used to identify “mechanisms” that are considered to be mediators of the observed effect	While slightly more broadly defined, this definition is compatible with probabilistic approaches to analysis	Causal analysis consists of identifying the configuration that links the outcome to mechanism(s) triggered by the context, often combining quantitative and qualitative data
Role given to theory	Theories in the form of universal laws can be deduced from empirical research (covering law principle)	Theories in the form of empirical knowledge derived from constant conjunction observations	Research contributes to developing theories of the middle range
Implications	Risk of reduction of mechanisms to measurable indicators, through which dynamic processes of change are reduced to correlations between variables that stand for more complex processes	In this view, and similar to definition 1, causation is reduced to the concatenation of elements in a causal chain. Causation is demonstrated to the degree that empirical regularities can identified	Empirical research allows investigation of a possible mechanism, thus identifying a plausible mechanism and may eventually lead to the identification of the actual mechanism. Research thus contributes to increasing the plausibility of the explanatory hypothesis

### Mediators and moderators or configurations?

Related to the conceptualization of “mechanism” is the analytical approach to identifying mechanisms and attributing effects to these entities. In several instances, Jamal et al. propose to use mediation analysis techniques to identify what mechanisms are at play. For instance, they write on p. 6: *“*In this stage, we will test hypotheses derived in stages 1 and 2 via quantitative analyses of effect mediation (to examine mechanisms) and moderation (to examine contextual contingencies).” These authors also write that “Causal mediation analysis helps to identify process or mediating variables that lie in the causal pathways between the treatment and the outcome….Mediators are post-baseline measures of interim effects which may or may not account for intervention effects on end-outcomes” (p. 6).

This explanation demonstrates the authors’ analytical strategy. To clarify how their approach is in contrast with, if not opposition to, the scientific realist approach, we turn to Mahoney [14]. This author presents three broad ways in which “mechanism” is defined in science (Table 2). First, when the term “causal mechanism” is used in experimental designs, it is mostly understood as a (set of) intervening variable(s) that explains why a correlation exists between an independent and dependent variable. Mechanisms are thus situated within the black box between independent and dependent variables and express themselves as a variable. “Yet, while the notion of mechanism as intervening process is useful, this definition unfortunately does not go beyond correlational assumptions” [14]), and thus, it yields little if any evidence on causation.

Mahoney identified a second definition of mechanism, which is “views causal mechanisms as mid-level theories or variables that can be used to explain a fairly wide range of outcomes” [14]. Here, causal mechanisms have been defined as “frequently occurring and easily recognizable causal patterns that are triggered under generally unknown conditions or with indeterminate consequences” [15], cited by Mahoney [14]. This definition focuses on the underlying theories of change and does not propose specific analytical strategies to demonstrate the effect of mechanism. Both the first and second definition of mechanism share a correlational analysis approach to causation that focuses on identifying antecedents regularly conjoined with outcomes (successionist mode of causal explanation).

A third definition is used by scientific realism, which considers a causal mechanism as *“*an unobserved entity that, when activated, generates an outcome of interest*”* [14] (p. 581). This is the generative causation view on mechanisms espoused by realist research, which holds that mechanisms are inherent properties of agents and structures. “Making and sustaining different choices requires a change in a participant’s reasoning (eg, in their values, beliefs, attitudes or the logic they apply to a particular situation) and/or the resources (eg, information, skills, material resources, support) they have available to them. This combination of reasoning and resources is what enables the programme to ‘work’ and is known as a ‘mechanism’” [11]. This generative conceptualisation moves the analysis of causation beyond correlational analysis.

Jamal et al. seem to have adopted the first definition that reduces mechanisms (and also “context”) to mere variables—although they are perhaps moving towards the second definition. In any case, their use of the terms “mediation” and “moderation” implies a variable-oriented approach to analysis in contrast to the configuration-oriented perspective that acknowledges complex causation adopted in scientific realism.

Jamal et al. extend the variable-oriented approach to the formulation of the hypotheses. The authors present how they developed a set of so-called pre-hypothesised intervention mechanisms (which they also label as mediation hypotheses) separately from a set of pre-hypothesised contextual barriers and facilitators. We can only assume the authors followed this pathway because it would be easier to statistically test the various hypotheses as separate strands. However, developing configuration-mechanism-outcome configurations entails more than “segmenting” the programme theory into a series of variables about context and another on dealing with what they call “intervention mechanisms”. In scientific realism, the explanation relies on showing the *relationship* between context and mechanism. Hypotheses should present a set of programme theories that explain how the outcome patterns can be explained by a configuration of intervention, actors, context and mechanisms. This reflects the acknowledgement of complex causation in realist evaluation.

This does not mean that realist research opposes the use of quantitative data. Realist research would rather not conceive of quantitative measures as “variables” (in the sense of things that vary in amount and cause subsequent variation in the next item in the equation) but as “indicators” (in the sense of a partial measure of an aspect of something that “indicates” whether it is present and/or the extent to which it is present). Using the latter definition, it is entirely possible to use quantitative measures for any of context (C), mechanism (M) or outcome (O), assuming of course that the C, M and O have been theorized previously, and that the indicators used are “fit for purpose” for that theory. This is a configurational usage of indicators as opposed to variable-based analysis.

In summary, in scientific realism, the analysis does not depend on assessing statistically the correlation between variables representing intervention, effect, moderators and mediators. Rather, the analysis uses whatever data and analytic methods appropriate to build, support, refute or refine plausible explanations that incorporate intervention, actors, outcomes, context and mechanisms. This brings us to our third way in which we consider scientific realism to differ from the methodology proposed by Jamal and colleagues.

### Can RCTs account for dynamic CMO configurations?

The final difference in the paper of Jamal et al. relates to the capacity of the RCT to unpack and make sense of the dynamic interplay among the intervention, actors, context and mechanisms that, from a realist perspective, contributes to the outcome patterns within a complex intervention. If one considers that CMO configurations (as proposed by Pawson and Tilley) can be assessed in a RCT, then this design should be able to demonstrate how and why outcome patterns are caused by mechanisms “triggered” in specific contexts. Here again, mechanisms are to be understood from the realist perspective and not as a chain of factors between the intervention and the outcome. The design should also be able to demonstrate how and why such CMO configurations vary for different people or in different contexts and change over time. Given the need for randomisation and control in an RCT, only relatively few and simple CMO configurations can be tested at a time. At best, then, the RCT may help us in assessing the relative contribution of mechanisms to outcome patterns if the causal configuration is uniform but not when it is likely that different mechanisms will generate different outcomes in different circumstances, as is the rule rather than the exception in any health intervention.

### Discussion

Jamal et al. write that “This paper provides the first guidance on the theoretical and methodological process of undertaking a realist RCT” (p. 7). We feel that this claim is somewhat premature. Not only has the primary study [16] that is the basis of this paper not been completed yet, the study was not designed as a realist study in the first place. More important, Jamal et al.’s definition and use of key realist concepts does not engage deeply enough with what we understand to be scientific realism. In particular, the different understanding of “mechanism” and the reliance on correlation between variables as the main analytical strategy squeezes realist analysis into the RCT design like a square peg into a round hole.

The core question concerns how far an approach based on definition of mechanisms 1 and 2 of Mahoney [14] can be combined with the realist definition of mechanism. From our point of view, these are incommensurable views. By its very reliance on randomization, control and use of variables and its roots in statistical analysis of correlations, we believe the development of RCTs to be realist according to Pawson and Tilley’s views is not fully feasible. We indeed fully agree with Byrne and Uprichard [17], who argue that cases are complex if causation works through interactive effects that in essence are based on interactions between people. If that is the case, Byrne and Uprichard argue, causal explanations require analysing interventions from a systems perspective with a case-based (i.e. configurational) and not a variable-based orientation.

## Conclusion

The paper by Jamal et al. is useful because it exposes the challenges of combining a realist evaluation approach (as developed by Pawson and Tilley) with the RCT design. However, the proposed approach does not allow testing theory in accordance to realist research principles. Not only does the proposed realist RCT design not deal with the RCT’s inherent inability to “unpack” complex interventions, it also does not enable the identification of the dynamic interplay between intervention, actors, context, mechanisms and outcomes that a configurational analysis demands. As a result, the proposed realist RCT design is not, as we understand it, genuinely realist in nature.

We might be accused of blocking possible innovation, but the guidance presented by Jamal et al. risks causing methodological confusion for researchers trying to understand realist research as espoused by Pawson and Tilley. This proposal for a realist RCT is indeed symptomatic of the mainstreaming of realist research into the wider health research and policy domains. It raises questions about the extent to which the RCT methodology can be modified to accommodate scientific realism. We showed that Jamal et al. consider mechanisms as observable, external treatments, and how their approach reduces complex causal processes to variables. We argue that their purportedly realist RCT design cannot provide a truly realist understanding, as it lacks compatibility with the analytical orientation needed to theorize and conduct realist research. As a consequence, it does not allow for plausible causal claims, nor does it produce warrants for transferable knowledge. This notwithstanding, we believe theory-*informed* RCTs would offer a way forward.

## Abbreviations

C, context; CMO, context-mechanism-outcome configuration; M, mechanism; O, outcome; RCT, randomised controlled trials; RE, realist evaluation
